# Artificial Intelligence Potential Impact on Resident Physician Education in Radiation Oncology

**DOI:** 10.1016/j.adro.2024.101505

**Published:** 2024-04-16

**Authors:** Neil D. Almeida, Rohil Shekher, Abigail Pepin, Tyler V. Schrand, Victor Goulenko, Anurag K. Singh, Simon Fung-Kee-Fung

**Affiliations:** a Department of Radiation Medicine, Roswell Park Comprehensive Cancer Center, Buffalo, New York; b Department of Radiation Oncology, Hospital of the University of Pennsylvania, Philadelphia, Pennsylvania; c Department of Chemistry, Bowling Green State University, Bowling Green, Ohio

## Introduction

Radiation oncology relies on computer software for digital processing and planning. Artificial intelligence (AI) employs computer algorithms to perform tasks including pattern recognition, decision making, and problem solving.^1^ AI has the potential to impact the quality of radiation therapy for cancer patients.^2^

AI promises to dramatically alter the medical educational landscape. Residents and fellows need to prepare for a future in which AI is integrated into their practice, as well as to understand the limitations of such technologies. Incorporation of AI and machine learning (ML) into residency curriculums is actively being addressed at some institutions to bolster traditional curriculums but is in its infancy.^3^

A survey of radiology residents demonstrated the discrepancy between the need for additional training and the desire.^4^ In that study, 83% of residents believed AI and ML education should be included in the curriculum. However, nearly a quarter of residents reported no AI curriculum at their institution. Despite this need, employment of AI in radiation oncology education has not been extensively explored in the literature. Herein, we investigated the impact of AI within radiation oncology, specifically the potential AI and ML hold to bolster resident physician education.

## AI in Contouring

Learning to contour relies on patients being seen as representative cases. However, there is significant variation among experts in the field for similar disease sites, which is further complicated by varied anatomy.^5^ AI can be employed to provide feedback on indexed cases as well as on variations acceptable for clinical learning. One multi-institutional clinical study used a deep learning-based contouring (DLC) system in patients with nasopharyngeal carcinoma. Three groups containing an expert and a beginner were tested with manual contouring and then a post-DLC-generated contour.^6^ DLC facilitated more performance improvement for organ at risk (OAR) contouring among the beginners than among the experts. Beginners also had significantly higher satisfaction rates than experts.

AI has been shown in multiple studies to improve auto-segmentation across disease sites. One study in head and neck cancer examined DLC and atlas-based auto-contouring (ABAS) in a manually contoured cohort. Compared with ABAS, DLC equaled or improved performance measures in 19 out of 22 OARs. DLC also significantly reduced delineation time compared with ABAS, was considered more precise, and was viewed more favorably.^7^ This points toward deep learning models being a useful adjunct in the education of junior residents in OAR contouring.

Auto-segmentation using deep learning models can also be helpful for residents learning target volumes in rare disease sites where limited data and institutional experience exist. Domoguen et al designed a deep learning architecture for the task of auto-segmenting nasopharyngeal carcinoma target volumes.^8^ Their study demonstrated that even with a small training data set, the proposed method improved target volume segmentations in patients with nasopharyngeal carcinoma compared with the current standard.

AI also has the potential to significantly improve the time spent delineating contours. One study investigating time saved for manual contouring versus atlas and DLC for OAR in lung cancer patients found a median time saved of 7.8 minutes for user-adjusted atlas-based contouring and 10 minutes for user-adjusted DLC.^9^ The user-adjusted DLC yielded a significant time reduction compared with the other groups. Chung et al investigated a trained deep learning-based auto-segmentation model for OARs and target volumes of patients with cervical cancer compared with manually drawn contours.^10^ Auto-segmentation reduced contouring time by 30 minutes in the same group of physicians. Notably, no auto-segmented contours had large errors, and the mean satisfaction score of the participants was 7 out of 10. Among the participating physicians from other institutions, the majority favored the auto-segmentation system in that study. As a resident physician progresses through their training in junior residency, with the time saved from manual contouring, their focus would shift to understanding contouring of subregions, such as cardiac substructures; reviewing contour expansions for accuracy; and demonstrating their ability to accurately assess AI contours before planning. Another study investigating the value added of automatic contouring tools used “the imitation game” (a variant of the Turing test) to determine whether observers could distinguish auto-contours from manually delineated contours. The misclassification rate was high for the thoracic OARs (esophagus, 30.0%; heart, 22.9%; left lung, 51.2%; right lung, 58.5%; mediastinum, 43.9%; and spinal cord, 46.8%).^11^ Inability to accurately judge the source of a contour indicates a reduced need for editing and therefore a greater time saving benefit overall.

Table 1 highlights commercially available AI-based auto-contouring tools that have emerged in recent years.

**Table 1:** AI-Driven Contouring Software

AI contouring software	Software workflow	Disease sites covered	Total structures advertised	Additional specification requirements
Mvision^26^	Cloud based software	Brain, breast, abdomen, thorax, male and female pelvis	>160 structures including OAR and target volume	None
MRCAT Prostate plus Auto-contouring^27^	MRI only based radiation therapy workflow	Primarily prostate, but additional software available for other disease sites including brain, pelvis, and head and neck	L&R Femoral heads, bladder (inner and outer wall), rectum, penile bulb, seminal vesicles, prostate, etc	Only compatible with Ingenia 1.5T and 3.0T MR-RT, Ambition 1.5T MR-RT, and Elition 3.0T MR-RT machines
Autocontour (Radformation)^28^	Stand-alone (downloadable) software	Chest and abdomen, head and neck, pelvis OAR, and lymph nodes on CT images and brain on MRI	>200 structures including OAR	none
OSAIRIS (Project InnerEye)^29^	Cloud based software	Head and neck and prostate treatment site structures	Up to 26 structures listed for H&N and prostate	none
RayStation (RaySearch)^30^	Deep learning auto-segmentation	Head and neck, thorax/abdomen, breast, lymph nodes, male pelvis	>70 structures on CT images	Should be installed on a higher end PC with a recommended screen resolution of 1920 × 1200 pixels (or 1920 × 1080); requires high speed GPU
MIM Software Contour ProtégéAI^31^	Cloud based software	Head and neck, thorax, lungs and liver, prostate and abdomen structures from CT images and prostate from MRI	>65 structures advertised	none
DLCExpert (Mirada Medical)^32^	Used on Mirada Medical's Workflow Box platform	Abdomen, breast, head and neck, prostate, and thorax	>160 structures advertised on CT and MRI images	Uses pre-existing treatment planning or image processing software
AI-Rad Companion Organs RT (Siemens Healthineers)^33^	Deployed through the Siemens teamplay digital health platform	Abdomen, head and neck, pelvis, and thorax	>60 structures	none
ART-Plan (TheraPanacea, Oncology Systems)^34^	Standalone software	Abdomen, brain, head and neck, thorax, and pelvis on CT images and abdomen, brain, and male pelvis on MRI	>150 structures	none
INTContour (Carina Medical)^35^	Standalone software	Abdomen, head and neck, male pelvis, and thorax	>60 structures	Can be integrated with Eclipse and RayStation softwares
Limbus Contour (Limbus AI, AMG Medtech)^36^	Locally hosted software	Lymph nodes, abdomen, breast, central nervous system, head and neck, lung, pelvis and prostate on CT images, and central nervous system, gynecologic and brachy structures on MRI	>160 structures	No need for cloud connection or GPU unit

*Abbreviations:* CT = computed tomography; GPU = graphics processing unit; H&N = head and neck; L&R = left and right; MRI = magnetic resonance imaging; OAR = organ at risk.

## Beam Planning

Resident education surrounding treatment planning and beam planning is highly variable. Assessing plans and determining areas to improve is based on experience. AI can provide residents with a more robust experience for beam planning and evaluation.

Placement of beams in 3-dimensional planning, intensity modulated radiation therapy (IMRT), and proton therapy is critical to avoid toxic side effects of radiation. Amit et al highlighted automatic learning-based beam angle selection for thoracic IMRT. The authors demonstrated that automated beam selection plans were acceptable compared with clinical plans and could potentially reduce the manual planning workload.^12^ Junior resident physicians could benefit from tools introducing the fundamentals of IMRT planning and assist with choosing specific beam angles. AI tools could prepare a junior resident physician to consider the number of fields used in an IMRT field and gain a deeper understanding of field arrangement and collimation. This not only improves their residency education but also prepares them for independent practice. Furthermore, Wang et al found that deep learning models can optimize beam direction and achieve the best possible spatial dose distribution.^13^ Junior residents can use such deep learning models to compare their own beam arrangements, thereby fine-tuning their skills and gaining a deeper understanding of dosimetry. In Table 1, we have summarized current resources and technologies for residency programs or residents who are interested in investigating these technologies independently.

Residency programs could implement assessments of an individual resident's understanding of the pitfalls of AI-based tools, including both beam planning and auto-contours. Resident physicians would first demonstrate their ability to individually assess the AI tools and understand the need to verify the accuracy of all critical structures before planning, particularly when high-dose or stereotactic radiation is used close to these structures. Resident physicians would have to be cognizant of the danger that lies in AI auto-contours and beam planning being unintentionally accepted without thorough validation. Furthermore, it is imperative for residents to recognize the importance of a systematic contour verification within the department. For instance, some facilities may initially mark auto-contour names with the label “ContourX-Prelim”, and the “-Prelim” is subsequently only redacted after review by the attending physician. Following this review, by both the resident and attending physician, dosimetry planners would ensure that no contours with the label “ContourX-Prelim” are used in the planning. The resident would ultimately be assessed throughout their training, as they move through each disease site, regarding their ability to not only accurately identify pertinent errors but also demonstrate an understanding of departmental contour review by the conclusion of their training.

Proton therapy is a rapidly evolving technology; however, proton centers are not available at every training institution. One study investigating the role of deep learning to assist in beam angle selection for proton therapy in patients with liver cancer found that AI-generated beam angles were comparable to human-selected beam angles.^14^ Such technologies could be useful to residents in understanding intricacies of proton beam planning, which is heavily dependent on the beam angles used to spare patients from potential toxicities.

In current clinical practice, utilization of the Quantitative Analyses of Normal Tissue Effects in Clinic (QUANTEC) is used to determine dose tolerance for OARs.^15^ However, utilization of ML to account for differences in radiomic features and patient-specific risk factors is on the horizon. In one such analysis, ML models performed better than normal tissue complication probability models in predicting xerostomia.^16^ Others have looked at employing such techniques to identify predictors for dysphagia and trismus, among others.^15^ Utilization of these technologies to allow for more individualized patient constraints can assist the beam-planning process to eliminate or minimize radiotoxic side effects of radiation therapy.

## Adaptive Radiation Therapy

Radiation therapy aims to optimize radiation to the target volume while minimizing radiation to surrounding OARs. Throughout the course of a patient's radiation course, the tumor volume and the patient's anatomy can change.^17^^,^^18^ Adaptive radiation therapy is a novel modality and active research area that aims to optimize the daily variation and customize the treatment to spare healthy tissue. Commercially available adaptive radiation therapy platforms, like the Varian Ethos system, employ AI-enhanced image segmentation and AI-driven treatment planning to optimize daily variation for treatment delivery. Day-to-day resident involvement in determining whether to proceed with pretreatment plans versus the adaptive plan will allow residents to gain experience in evaluating plans in real time and assess trade-offs. More data are needed in terms of whether this technology's improvement in OAR constraints and coverage improves clinical outcomes.

ML applications could be used to help residents evaluate a plan's clinical acceptability. McIntosh et al investigated the integration of ML in prostate cancer, where treating physicians assessed ML and human-generated radiation therapy treatment plans in a blinded manner. ML-based radiation therapy treatment plans were faster than human-driven plans and clinically acceptable in 89% of cases.^19^ Another study investigating autogenerated pretreatment plans for pelvic cases reported similar target volume coverage and OAR doses compared with their institution reference, with 75% of AI-segmentations requiring no or minor editing.^20^ As residents progress through their training, autogenerated pretreatment plans could be used to assess residents' understanding of AI pretreatment plan pitfalls.

## Brachytherapy

Similar to its utility in external beam radiation therapy, AI holds great promise in brachytherapy applications. Nicolae et al compared the preimplantation treatment planning time of an ML model with that of a brachytherapist (BT) in prostate low-dose-rate brachytherapy. The authors reported that the ML model significantly reduced treatment planning times, compared with BT-generated plans. Additionally, there was no dosimetric difference between ML and BT treatment plans, with the average prostate V_150%_ being 4% lower for ML plans.^21^ Another study demonstrated the noninferiority of ML-generated treatment plans compared with BT-generated treatment plans in prostate brachytherapy. Patients were 1:1 randomized to receive an ML-based planning system versus a manual technique, with no significant difference in the similarity of each modality or degree of modification needed for treatment. However, the ML-based treatment plan had a significantly reduced planning time.^21^

AI can be applied to additional aspects of the brachytherapy treatment, including allowing clinicians to localize and assess the implant quality.^22^^,^^23^ Marcrom et al reported that among United States radiation oncology residents, caseload in BT was the greatest perceived barrier in training, with confidence correlating with case volume.^24^ Additionally, Lichter et al reported that lack of exposure during resident physician training, low volume treatment centers, and financial constraints contribute to underutilization of brachytherapy in the United States.^25^ AI tools promise to fill this void by supplementing a program's caseload limitations, bolstering institutions with limitations in advanced imaging techniques, and ultimately increasing resident confidence in brachytherapy and cervical cancer treatment paradigms.

## Conclusion

AI is rapidly immersing itself in all aspects of health care and is slowly becoming rooted in the radiation therapy treatment steps. As both imaging modalities and radiation treatment devices continue to advance alongside AI, understanding how these facets interplay will be paramount. This review has shown that AI can excel in multiple aspects of the radiation therapy process and promises to not only improve the quality of resident education, but also familiarize residents with a vital resource becoming increasingly prevalent in medicine.

## Disclosures

The authors declare that they have no known competing financial interests or personal relationships that could have appeared to influence the work reported in this paper.
